# Multiple Partial Regularized Nonnegative Matrix Factorization for Predicting Ontological Functions of lncRNAs

**DOI:** 10.3389/fgene.2018.00685

**Published:** 2019-01-23

**Authors:** Jianbang Zhao, Xiaoke Ma

**Affiliations:** ^1^College of Information Engineering, Northwest Agriculture & Forestry University, Xianyang, China; ^2^School of Computer Science and Technology, Xidian University, Xi'an, China

**Keywords:** lncRNA, nonnegative matrix factorization, gene ontology, networks, regularization

## Abstract

Long non-coding RNAs (LncRNA) are critical regulators for biological processes, which are highly related to complex diseases. Even though the next generation sequence technology facilitates the discovery of a great number of lncRNAs, the knowledge about the functions of lncRNAs is limited. Thus, it is promising to predict the functions of lncRNAs, which shed light on revealing the mechanisms of complex diseases. The current algorithms predict the functions of lncRNA by using the features of protein-coding genes. Generally speaking, these algorithms fuse heterogeneous genomic data to construct lncRNA-gene associations via a linear combination, which cannot fully characterize the function-lncRNA relations. To overcome this issue, we present an nonnegative matrix factorization algorithm with multiple partial regularization (aka MPrNMF) to predict the functions of lncRNAs without fusing the heterogeneous genomic data. In details, for each type of genomic data, we construct the lncRNA-gene associations, resulting in multiple associations. The proposed method integrates separately them via regularization strategy, rather than fuse them into a single type of associations. The results demonstrate that the proposed algorithm outperforms state-of-the-art methods based network-analysis. The model and algorithm provide an effective way to explore the functions of lncRNAs.

## 1. Introduction

Long non-coding RNAs (lncRNAs) are a type of non-coding RNAs with more than 200 nucleotides in length, which have very little or no potential to encode proteins (Mercer et al., 2009). In the past lncRNAs are categorized as “dark matter” and “junks.” However, more and more evidence demonstrates that lncRNAs are critical regulators for biological processes, such as immune response, cell development and differentiation, as well as gene imprinting (Morris and Mattick., 2014; Turner et al., 2014; Ma et al., 2017). Furthermore, lncRNAs are highly related to diseases and cancers (Zou et al., 2015, 2016; Zhu et al., 2018). Largely due to the high-throughput biological techniques, particularly the next generation sequence (NGS), large numbers of lncRNAs have been identified (Iyer et al., 2015; Fang et al., 2018).

Compared to the protein-coding genes (genes for short), the functions of vast majority of lncRNAs are unknown. Thus, it is promising to predict the functions of lncRNAs, which are critical for revealing the underlying mechanisms of gene regulation. The approaches for annotating the functions of lncRNAs are classified into two classes: the biological experiment and computational based methods. Currently, the functions of some lncRNAs are validated by the biological experiment based methods. For example, based on the RNA-sequencing data, the mechanistic analysis reveals that UCA1 physically interacts with PTBP1 and ALAS2, which stabilizes ALAS2 (Liu et al., 2018). Li et al. (2016) utilized the RT-PCR to detect the expression profiles of lncRNA TUG1 in glioma, and found that TUG1 is involved in the apoptosis and cell proliferation. Based on the cap analysis of gene expression (CAGE) data, FANTOME generated a comprehensive atlas of 27919 human lncRNA genes across 1829 samples from the major human primary cell types and tissues (Hon et al., 2017). Wang et al. (2018) identified the function of NEAT1 using the enhanced green fluorescent protein reporter in human cells.

Except the expression profiles, some lncRNAs execute their functions via interacting with other bio-molecules, such as DNAs, RNAs and proteins. Mercer and Mattick (2013) focused on the lncRNAs as epigenetic modulators via binding to chromatin-modifying proteins and recruiting their catalytic activity to specific sites in the genome. Efforts is devoted to investigate the lncRNA-DNA interactions, including the chromatin isolation by RNA purification (Chu et al., 2012; Nowak et al., 2014). Furthermore, Ferre et al. (2016) identified the protein-lncRNA interactions, offering essential clues for a better understanding of lncRNA cellular mechanisms and their disease-associated perturbations.

Even though the experiment based approaches for the functions of lncRNAs are reliable, they are criticized by the expensive cost and complicated operations. Thus, the computational algorithms for the prediction of lncRNA functions provide an alternative, which become more and more important. Based on the assumption that the molecules with the same or similar functions have the same or similar patterns. Some efforts explore the co-expression patterns (Lee et al., 2004; Necsulea et al., 2014). Furthermore, the gene set enrichment analysis (GSEA) based on the statistics is also adopted to identify the functions of lncRNAs (Guttman et al., 2009). To explore the knowledge from genes, (Liao et al., 2011) combined the expression profiles of lncRNAs and genes to construct a coding and non-coding gene co-expression network according to the expression profiles in the GEO database, then predicted the functions of more than 300 mouse lncRNAs based on the co-expression modules. In order to make use of the global information, Guo et al. (2013) constructed a bi-colored network via integrating the expression profiles of lncRNA and genes, then provided the lnc-GFP algorithm to predict the functions of lncRNAs. Jiang et al. (2015) employed the statistical test to annotate the functions of lncRNAs. Recently, Zhang et al. (2018) proposed the NeuralNetL2GO algorithm, which uses neural networks to annotate lncRNAs.

Actually, there are many different genomic data to link the lncRNA and genes, for example gene co-expression, connection to the diseases, protein binding sites. The current algorithms integrate multiple heterogeneous genomic data into a single network via weighted or unweighted linear functions, which are criticized for not fully characterizing the links between lncRNAs and genes. Evidence shows that the linear combination destroys the patterns in the integrated network (Ma and Dong, 2017; Ma et al., 2019). In fact, each type of genomic data provides a perspective of the links between lncRNAs and genes. The ultimate goal of this study is to provide a computational method to predict functions of lncRNAs by fusing heterogeneous data. As shown in **Figure 2**, we construct multiple bi-color networks for lncRNAs and genes. Then, the multiple partial regularized nonnegative matrix factorization (MPrNMF) algorithm is proposed to simultaneously factorize the multiple networks. In order to improve the accuracy, the regularization strategy is adopted, where the factorized feature matrix preserves the links between lncRNAs and genes. The results demonstrate that the proposed method outperforms these algorithms based on the single bio-colored network, implying the proposed method is promising.

The rest of this paper is organized as: section 2 briefly reviews the related works on the prediction of lncRNAs functions. Section 3 describes the procedure of the proposed method. Section 4 shows the experimental results. Finally, the conclusion is presented in section 5.

## 2. Related Works

In this section, we first introduce the mathematical notations that are widely used in the forthcoming sections. Then, we review state-of-the-art methods for the prediction of lncRNA functions.

### 2.1. Notations

The notations are summarized in Table 1. Let *n* be the number of entities in the networks. Generally speaking, let *n*_*o*_ be the number of ontological functions in Gene Ontology (GO), *n*_*g*_ be the number of proteins (genes) in the PPI network, *n*_*l*_ be the number of lncRNAs in the co-expression network. Let *G*_*g*_, *G*_*l*_ be the PPI and lncRNA co-expression networks, respectively. The adjacency matrix for *G*_*g*_, denoted by *W*_*g*_, corresponds to a *n*_*g*_×*n*_*g*_ matrix whose element $w_{ij}^{\left[g\right]}$ is the weight on edge (*v*_*i*_, *v*_*j*_) in *G*_*g*_. The degree of vertex *v*_*i*_ in *G*_*g*_ is the sum of weights on edges connecting *v*_*i*_, i.e., $d_{i}^{\left[g\right]}=\underset{j}{\sum }w_{ij}^{\left[g\right]}$. The degree matrix *D*_*g*_ is the diagonal matrix with degree sequence of *G*_*g*_, i.e., $D_{g}=diag\left(d_{1}^{\left[g\right]},d_{2}^{\left[g\right]},\ldots ,d_{n}^{\left[g\right]}\right)$. The Laplacian matrix of *G*_*g*_ is defined as $L_{g}=I-D_{g}^{-1/2}W_{g}D_{g}^{-1/2}$. Analogously, the adjacent matrix of *G*_*l*_ is denoted by *W*_*l*_. Let *L*_*l*_ be the Laplacian matrix for *G*_*l*_. The associations between heterogeneous entities are denoted by matrix. Specifically, let *X* be the known lncRNA-ontology associations, *Y* be the known gene-lncRNA associations, and *Y*_1_(*Y*_2_) be the known lncRNA-disease (gene-disease) associations, respectively.

**Table 1:** Notations and descriptions.

**Symbol**	**Definition and description**
*n*_*o*_, *n*_*g*_, *n*_*l*_	Number of ontological functions, genes and lncRNAs
*G*	graph with vertex set *V* and edge set *E*
*X*	Known lincRNA-ontology associations
*Y*_1_, *Y*_2_	Known lncRNA-gene associations
*G*_*g*_	Protein-Protein interaction (PPI) network
*G*_*l*_	LncRNA co-expression network
${\bar{W}}_{g}$	Normalized adjacent matrix of the PPI network ${\bar{W}}_{g}=D^{-1/2}W_{g}D^{-1/2}$
${\bar{W}}_{l}$	Normalized adjacent matrix of lncRNA co-expression network ${\bar{W}}_{g}=D^{-1/2}W_{l}D^{-1/2}$
*L*_*g*_	Normalized Laplacian matrix of *G*_*g*_, i.e., $L_{g}=I-{\bar{W}}_{g}$
*L*_*l*_	Normalized Laplacian matrix of *G*_*l*_, i.e., $L_{l}=I-{\bar{W}}_{l}$

### 2.2. Related Algorithms

**Figure 1:**
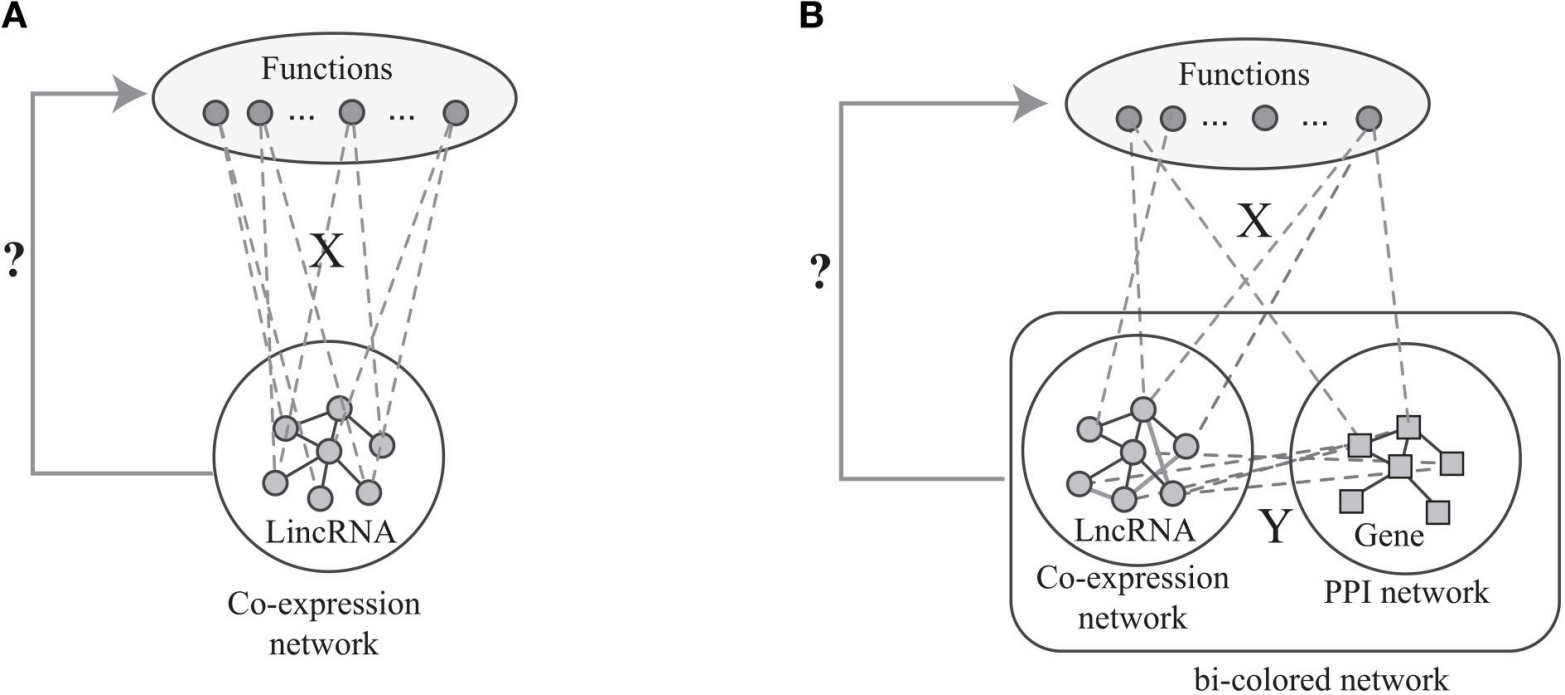
The flowchart of the current algorithms based on network analysis: **(A)** label propagation method based on the lncRNA co-expression network, **(B)** label propagation method based on the bio-colored network.

The label propagation algorithm is successfully applied to predict phenotype-gene associations with various backgrounds (Li and Patra, 2010; Vanunu et al., 2010), where the principle of the label propagation algorithms is illustrated in Figure 1A. In details, label propagation assumes that the well connected lncRNAs in *G*_*l*_ are very likely to be the same label, which leads to the following objective function

(1) $$\begin{array}{r} J_{LP}=\theta tr\left(\hat{X}L_{l}{\hat{X}}^{{}^{\prime }}\right)+\left(1-\theta \right)||\hat{X}-X|{|}^{2}, \end{array}$$

where $\hat{X}$ is the predicted lncRNA-ontology associations, θ ∈ (0, 1) is the parameter controlling the contributions of two terms in Equation (1), *tr*(*A*) is the trace of matrix *A*, i.e., $tr\left(A\right)=\underset{i}{\sum }a_{ii}$ and ||*A*|| is the *l*_2_ norm of matrix *A*. In Equation (1), the first item characterizes how the predicted lncRNA-ontology associations $\hat{X}$ is consistent with the lncRNA co-expressed network, while the second one measures the good the predicted associations fit the initial labeling.

However, the number of predicted associations is largely determined by the sparsity of the known associations in *X*. When *X* is very sparse, the number of predicted associations is limited. Actually, *X* is very sparse since the GO functions of vast majority of lncRNAs are unknown. Fortunately, the GO functions of most proteins are known. Thus, the available algorithms overcome this limitation of the label propagation algorithm via integrating the proteins and lncRNAs as shown in Figure 1B. Specifically, given the known protein-GO associations *X*, PPI network *G*_*g*_, lncRNA co-expression network *G*_*l*_ and lncRNA-gene associations *Y*, the ultimate goal is to predict the lncRNA-ontology associations via integrative analysis of heterogeneous data. The lnc-GFP algorithm (Guo et al., 2013) follows the label propagation method by using the bi-colored network, which is defined as

(2) $$\begin{array}{r} C=\left[\begin{array}{cc} W_{l} & Y\\ Y^{{}^{\prime }} & W_{g} \end{array}\right]. \end{array}$$

Thus, the objective function in Equation (1) is transformed into

(3) $$\begin{array}{r} J_{LP}=\theta tr\left(\hat{X}L_{C}{\hat{X}}^{{}^{\prime }}\right)+\left(1-\theta \right)||\hat{X}-X|{|}^{2}, \end{array}$$

where *L*_*C*_ is the Laplacian matrix of the bi-colored network *C*. The KATZLGO method (Zhang et al., 2017) predicts the GO functions of lncRNAs by using the KATZ score of the bi-colored network, which counts the paths with various lengths in the bi-colored networks.

The bi-colored based methods make use of lncRNA-gene associations to predict the functions of lncRNAs. To explore the knowledge in *G*_*l*_ and *G*_*g*_, Petergrosso et al. (2017) proposed the dual label propagation (DLP) to predict the phenotome-genome associations. Specifically, the objective function in Equation(1) based on the DLP model can be re-written as

(4) $$\begin{array}{r} J_{DLP}=||\hat{X}-X|{|}^{2}+\beta tr\left(\hat{X}L_{g}{\hat{X}}^{{}^{\prime }}\right)+\gamma tr\left(\hat{X}L_{l}{\hat{X}}^{{}^{\prime }}\right), \end{array}$$

where β ≥ 0, γ ≥ 0 are tuning parameters. The first item measures the consistence between the predicted associations and the bi-colored network, and the last two ones measures the smoothness in the PPI and lncRNA networks.

Most of the available algorithms for the prediction of LncRNA functions are based on the bi-colored network model. In this study, we investigate the possibility to predict the functions of lncRNAs via integrating multiple networks, where each type of genomic data is used to construct the lncRNA-gene associations.

## 3. Methods

**Figure 2:**
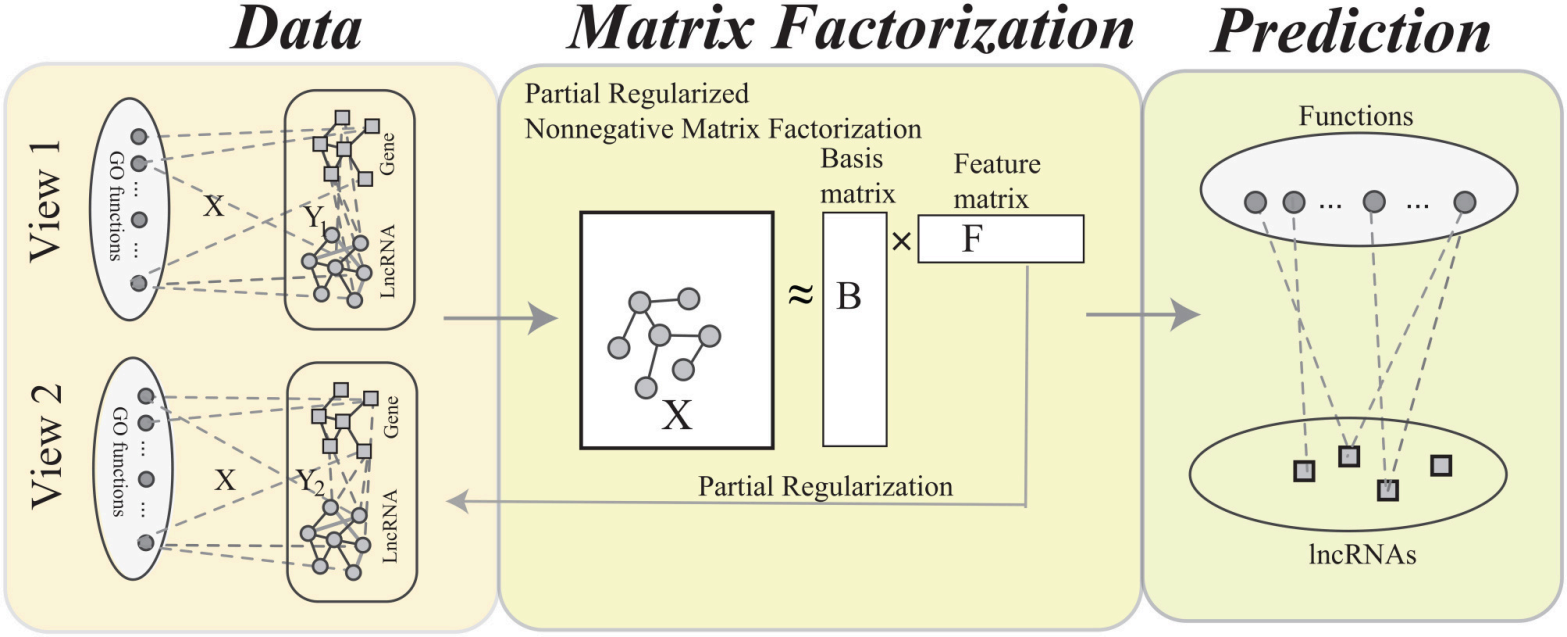
The flowchart of the MPrNMF algorithm, which consists of three components: network construction, matrix factorization and function prediction. In the network construction, each type of heterogenous lncRNA-gene associations is used to construct a bi-colored network. The matrix factorization procedure obtains approximation of lncRNA(gene)-ontology associations *X*, where the feature matrix *F* reflects multiple lncRNA-gene associations. The function prediction procedure is based on the decomposed matrices.

The procedure of MPrNMF is illustrated in Figure 2. In this section, we derive the objective function and optimizing rules of the proposed algorithm in turns.

### 3.1. Objective Function

All these bi-colored network based algorithms predict the lncRNA-ontology associations based on the single bi-colored network via integrating various genomic data. In this study, we construct two bi-colored networks, where each one corresponds to a view of the lncRNA-gene associations. In the first one, the lncRNA-gene associations are determined by the pearson correlation coefficient between the expression profiles of lncRNAs and genes. And, the second lncRNA-gene associations are determined by the diseases. In details, the lncRNA-gene association is the Jaccard index of the diseases related to lncRNAs and genes. The *i*-th view of the bi-colored network is denoted by

(5) $$\begin{array}{r} C_{i}=\left[\begin{array}{cc} W_{l} & Y_{i}\\ Y_{i}^{{}^{\prime }} & W_{g} \end{array}\right], \end{array}$$

where *Y*_*i*_(*i* = 1, 2) is the lncRNA-gene associations in the *i*-th view.

Given the lncRNAs(genes)-ontology associations *X*, NMF aims at obtaining approximation of *X* via the product of two nonnegative matrices *B*_1_ and *F*_*t*_ (Lee and Seung, 1999), i.e.,

(6) $$\begin{array}{r} J=||X-BF|{|}^{2},\;s.t.\;B\geq 0,F\geq 0, \end{array}$$

where *B* is the basis matrix and *F* is the feature matrix. Furthermore, we also expect the feature matrix *F* also reflects the topological structure of multiple views of the bi-colored network, which is implemented via the regularization. To this end, the Equation (6) is reformulated as

(7) $$\begin{array}{r} J=||X-BF|{|}^{2}+\alpha \overset{2}{\underset{i=1}{\sum }}tr\left(FC_{i}F^{{}^{\prime }}\right),\;s.t.\;B\geq 0,F\geq 0, \end{array}$$

where parameter α controls the importance of the regularization items and *tr*(*A*) is the trace of matrix *A*, i.e., $tr\left(A\right)=\underset{i}{\sum }a_{ii}$.

In the bi-colored network, the vertices consist of lncRNAs and genes. Thus, the feature matrix *F* is also re-written as *F* = [*F*_*l*_, *F*_*g*_], where *F*_*l*_ denotes the part for the lncRNAs and *F*_*g*_ for genes. Thus, $tr\left(FC_{i}F^{{}^{\prime }}\right)$ is reformulated as

$$\begin{array}{l} tr(FC_{i}F^{{}^{\prime }})=\left(tr[F_{l},F_{g}]\left[\begin{array}{cc} W_{l} & Y_{i}\\ Y_{i}^{{}^{\prime }} & W_{g} \end{array}\right]\left[\begin{array}{c} F_{l}^{{}^{\prime }}\\ F_{g}^{{}^{\prime }} \end{array}\right]\right)\\ \;=tr(F_{g}W_{g}F_{g}^{{}^{\prime }}+F_{l}Y_{i}^{{}^{\prime }}F_{g}^{{}^{\prime }}+F_{l}Y_{i}F_{g}^{{}^{\prime }}+F_{l}W_{l}F_{l}^{{}^{\prime }})\\ \;=tr(F_{g}W_{g}F_{g}^{{}^{\prime }})+2tr(F_{l}Y_{i}^{{}^{\prime }}F_{g}^{{}^{\prime }})+tr(F_{l}W_{l}F_{l}^{{}^{\prime }}). \end{array}$$

The above equation indicates that the regularization item for the bi-colored network can be divided into three components: *W*_*g*_, *W*_*l*_ and *Y*_*i*_. In the two views, the only difference is the lncRNA-gene relations. Thus, we expect the regularization item can fully relect the lncRNA-gene relations *Y*_*i*_. In this case, the objective function in Equation (7) is transformed into

(8) $$\begin{array}{rcl} \min J & = & ||X-BF|{|}^{2}+\alpha \overset{2}{\underset{i=1}{\sum }}tr\left(F_{l}Y_{i}F_{g}^{{}^{\prime }}\right) \end{array}$$

$$\begin{array}{rc} s.t. & B\geq 0,F\geq 0,F^{{}^{\prime }}1_{n_{l}+n_{g}}=1_{n_{l}+n_{g}} \end{array}$$

where 1_*n*_ is the column vector with all elements 1. The *l*_1_-norm constraint on matrix *F*_*t*_ is adopted to obtain sparsity solutions.

### 3.2. Optimization Rules

To optimize the objective function in Equation (8), we derive the updating rules for matrix *B* and *F*. Since the objective function is non-convex, we update one matrix by fixing the other, which continues until the termination criterion is reached.

By integrating the sparsity constraint of matrix *F*, the Lagrange function for objective function is formulated as

$$\begin{array}{l} L\;=\;\|X-BF{\|}^{2}+2\alpha \sum_{i=1}^{2}tr(F_{g}Y_{i}F_{l}^{\prime })+tr(\Lambda (F^{\prime }1_{n_{l}+n_{g}}-1_{n_{l}+n_{g}})\\ \;\;\;\;\;\;\;\;\;\;\;\;(F^{\prime }{}^{1_{n_{l}+n_{g}}-1_{n_{l}+n_{g}})\prime })\\ \;\;\;\;=\;\|X-BF{\|}^{2}+\;\alpha \sum_{i=1}^{2}tr(FC_{i}^{*}F^{{}^{\prime }})+tr(\Lambda (F^{{}^{\prime }}1_{n_{l}+n_{g}}-1_{n_{l}+n_{g}})\\ \;\;\;\;\;\;\;\;\;\;\;\;\left(F^{\prime }1_{n_{l}+n_{g}}-1_{n_{l}+n_{g}})\prime \right), \end{array}\;$$

where matrix $C_{i}^{*}$ is defined as

$$\begin{array}{l} C_{i}^{*}=\left[\begin{array}{cc} 0 & Y_{i}\\ Y_{i}^{{}^{\prime }} & 0 \end{array}\right]. \end{array}$$

The derivative of *L* on *B* is calculated as

$$\begin{array}{l} \frac{1}{2}\nabla_{B}L=XF^{{}^{\prime }}-BFF^{{}^{\prime }}, \end{array}$$

and the derivative of *L* on *F* is written as

$$\begin{array}{l} \frac{1}{2}\nabla_{F}L=B^{{}^{\prime }}X-B^{{}^{\prime }}BF^{{}^{\prime }}+\alpha \overset{2}{\underset{i=1}{\sum }}FC_{i}^{*}-1_{n_{l}+n_{g}}1_{n_{l}+n_{g}}^{{}^{\prime }}\Lambda . \end{array}$$

According to the Karush-Kuhn-Tucker condition, by setting $\frac{1}{2}\nabla_{B}L$=0, we obtain the updating rule for matrix *B* as

(9) $$\begin{array}{r} B=B⊙\sqrt{\frac{\left[BFF^{{}^{\prime }}\right]}{\left[XF^{{}^{\prime }}\right]}}, \end{array}$$

where ⊙ denotes element-wise product, [·]/[·] denotes element-wise division and $\sqrt{\cdot }$ is the element-wise square root. Analogously, the updating rule for matrix *F* is derived as

(10) $$\begin{array}{r} F=F⊙\sqrt{\frac{\left[B^{{}^{\prime }}BF^{{}^{\prime }}\right]}{\left[B^{{}^{\prime }}X\;+\;\alpha \left(FC_{1}^{*}\;+\;FC_{2}^{*}\right)\right]}}. \end{array}$$

After obtaining matrices *B* and *F*, we divide the matrix *B* = $\left[\begin{array}{c} B_{l}\\ B_{g} \end{array}\right]$. The prediction of lncRNA-ontology is obtained as *B*_*l*_*F*_*l*_. The procedure of the proposed algorithm is illustrated in Algorithm 1. Usually, the number of iterations is 100.

**Algorithm 1:** The MPrNMF algorithm

**Input:**
*Y*_*i*_(1 ≥ *i* ≥ *n*): The multiple views of lncRNA-gene associations;
*X*: The known lncRNA(gene)-ontology associations;
*k*: number of communities;
α: weight for multiple views;
**Output:**
${\hat{X}}_{l}$: the predicted lncRNA-ontology associations.
***Part I: Matrix Decomposition***
1: Initialing randomly *B* and *F*;
2: Fixed matrix *F*, update matrix *B* according to Equation (9);
3: Fixed matrix *B*, update matrix *F* according to Equation (10);
4: Continue Step 2 and 3 until the termination criterion is reached;
***Part II: Predicting lncRNA-ontology associations***
5: Predicting the lncRNA-ontology associations as ${\hat{X}}_{l}=B_{l}F_{l}$;
6: **return** ${\hat{X}}_{l}$.

## 4. Results

### 4.1. Data

The PPI network is downloaded from the BioGrid database (https://thebiogrid.org/). We select the maximal connected subgraph in the PPI network for analysis. The lncRNAs are downloaded from the GENCODE database (https://www.gencodegenes.org/). The gene-disease associations are downloaded from the OMIM database (https://omim.org/), while the lncRNA-disease associations are downloaded from the LncRNADisease database (http://www.cuilab.cn/lncrnadisease). The expression profiles are downloaded from the COXPRESdb database Okamura et al. (2018) (http://coxpresdb.jp/), where the three preprocessed datasets, including Hsa.c4-1, Hsa2.c2-0, and Hsa3.c1-0, are used.

Since there is no available public database for the ontology of lncRNAs, Zhang and Ma (2018) manually curate a set of 55 lncRNAs with 129 GO terms by literature searching. We adopt this dataset as benchmark to test the performance of the proposed method.

### 4.2. Criterion

To predict the lncRNA-ontology associations, the output of the proposed algorithm is a real value in the interval [0,1]. Hence a threshold is need to determine the final prediction. Following the NeuraNetL2GO algorithm (Zhang and Ma, 2018), we use the Recall, Precision and Fmax to quantify the accuracy of algorithms. Specifically, let *t* be the threshold, and *P*(*t*) be the set of predicted ontology, and *T* be the ontology in the benchmark dataset. For the *i*-th lncRNA, the true positives (TP), false positives (FP) and false negatives (FN) are defined as

(11) $$\begin{array}{r} TP_{i}=\underset{o\in O}{\sum }I\left(f\in P_{i}\left(t\right)\wedge f\in T_{i}\right), \end{array}$$

(12) $$\begin{array}{r} FP_{i}=\underset{o\in O}{\sum }I\left(f\in P_{i}\left(t\right)\wedge f\notin T_{i}\right), \end{array}$$

(13) $$\begin{array}{r} FN_{i}=\underset{o\in O}{\sum }I\left(f\notin P_{i}\left(t\right)\wedge f\in T_{i}\right), \end{array}$$

where *o* is an ontology, O denotes the set of all functions, and *I*(*x*) is indicator function with value 1 if *x* is true, 0 otherwise. The recall, precision, and Fmax are defined as

(14) $$\begin{array}{r} Recall=\frac{\sum_{i}TP_{i}}{\sum_{i}TP_{i}\;+\;\sum_{i}FN_{i}}, \end{array}$$

(15) $$\begin{array}{r} Precision=\frac{\sum_{i}TP_{i}}{\sum_{i}TP_{i}\;+\;\sum_{i}FP_{i}}, \end{array}$$

(16) $$\begin{array}{r} Fmax=\underset{t}{\max}\frac{2Recall\left(t\right)Precision\left(t\right)}{Recall\left(t\right)\;+\;Precision\left(t\right)}. \end{array}$$

### 4.3. Parameter Selection

There are two parameters involved in MPrNMF: parameter *k* is the number of features, and parameter α controls the relative importance of partial regularization items. On the parameter *k*, Wu et al. (2016) proposed the instability based NMF model for parameter selection. For each *k*, MPrNMF runs τ times with random initial solutions and obtains τ basis matrices, denoted by *B*_1_, …, *B*_τ_. Given two matrices *B*_1_ and *B*_2_, a τ × τ matrix *H* is defined where the element *h*_*ij*_ is the cross correlation between the *i*-th column of matrix *B*_1_ and the *j*-th column of matrix *B*_2_. The dissimilarity between *B*_1_ and *B*_2_ is defined as

$$\begin{array}{l} diss\left(B_{1},B_{2}\right)=\frac{1}{2k}\left(2k-\underset{j}{\sum }\max H_{.j}-\underset{i}{\sum }\max H_{i.}\right), \end{array}$$

where *H*_.*j*_ denotes the *j*-th column of matrix *Q*. The instability is the discrepancy of all the basis matrices for *k*, which is defined as

$$\begin{array}{l} \Upsilon \left(k\right)=\frac{2}{\tau \left(\tau -1\right)}\underset{1\leq i<j\leq \tau }{\sum }diss\left(B_{i},B_{j}\right). \end{array}$$

**Figure 3:**
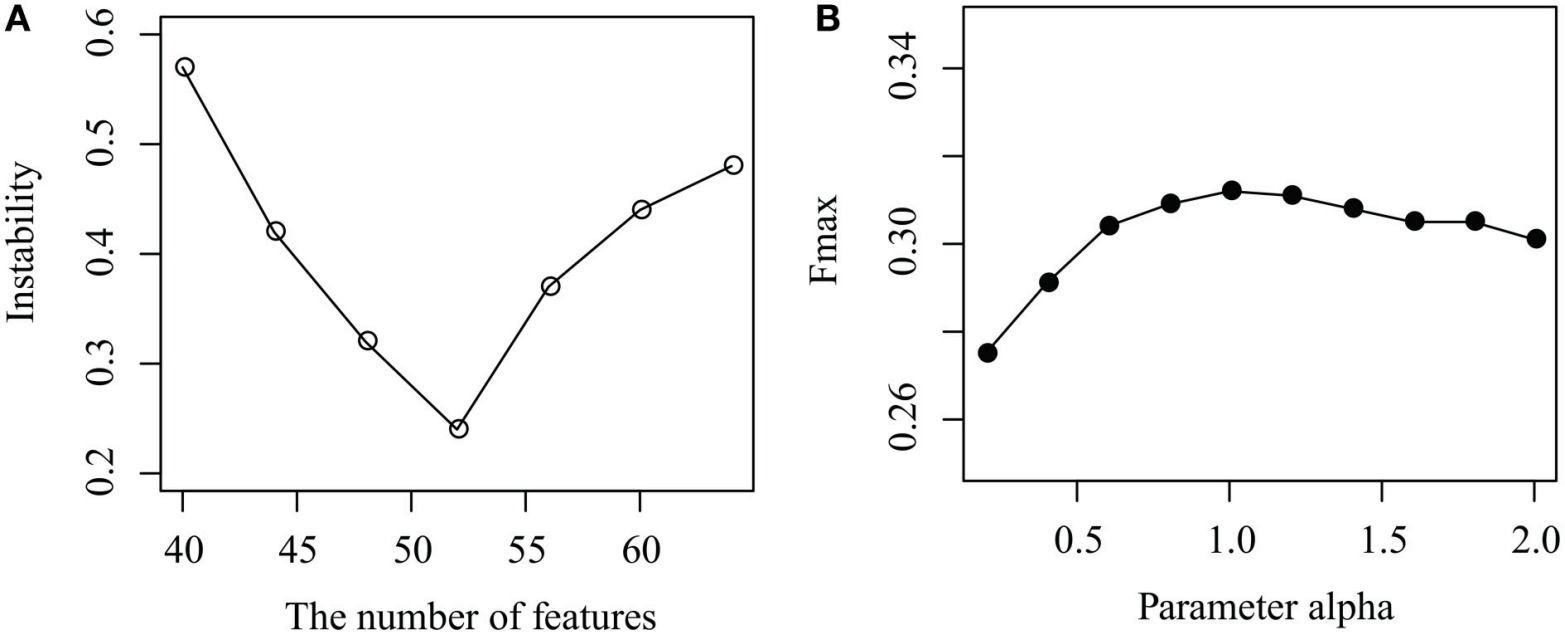
Parameter selection: **(A)** how instability changes as the number of features *k* increases, and **(B)** how the parameter α effects the performance of the proposed algorithm.

As shown in Figure 3A, the instability of MPrNMF changes as the number of features *k* ranges from 40 to 64 with gap 4. When *k* < 52, the instability decreases, while it increases if *k*>52. The reason is that when *k* is small, the number of features cannot fully characterize topological structure of associations, while large *k* results in the redundance of features. It reaches minimum at *k* = 52. Thus, we set *k* = 52.

How the parameter α effects the performance of MPrNMF is illustrated in Figure 3B, where the Fmax changes as α increases from 0.1 to 2 with a gap 0.2. It is easy to assert that, when α increases from 0.1 to 1, the performance also improves. The accuracy of the proposed algorithm is robust when α > 1. The reason is that when α is small, the objective function is dominated by the associations between lncRNA(gene)-ontology diseases. As α increases, the contribution of the regularization items for the multiple views of lncRNA-gene associations increases, improving the accuracy. Therefore, we set α = 1 since it reaches a good balance between lncRNA(gene)-ontology associations and lncRNA-gene associations.

### 4.4. Performance

To fully validate the performance of MPrNMF, three algorithms are selected for a comparison, including lnc-GFP (Guo et al., 2013), Lnc2Function (Jiang et al., 2015) and NeuraNetL2GO (Zhang and Ma, 2018, because of their excellent performance. In this study, we only focus on the biological process of GO terms.

**Figure 4:**
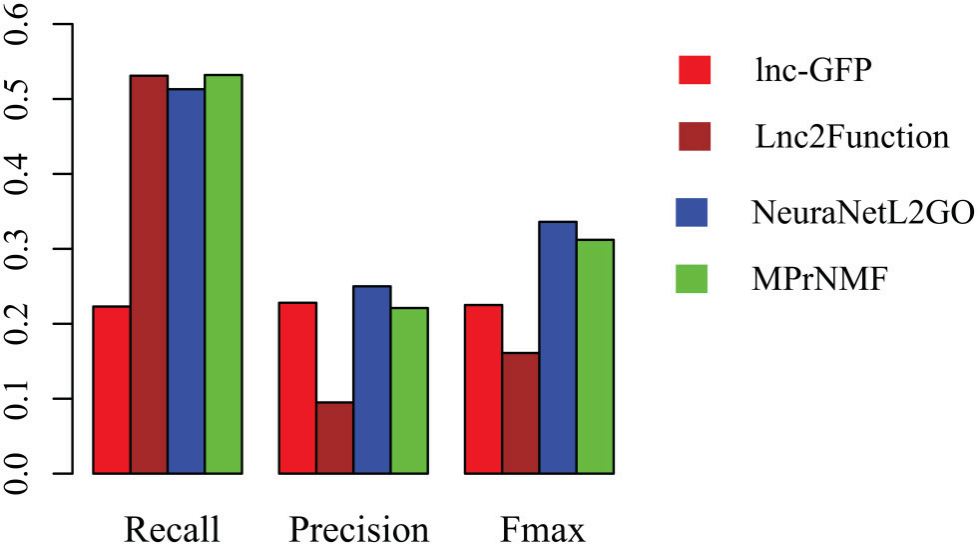
The performance of various algorithms on the prediction of ontology of lncRNAs in terms of Recall, Precision and Fmax.

The accuracy of various algorithms is shown in Figure 4, where recall, precision and Fmax are adopted for measuring the performance. These result demonstrate that: (i) MPrNMF achieves the best performance on the recall; (ii) MPrNMF outperforms the lnc-GFP and Lnc2Function; (iii) MPrNMF is inferior to the NeuraNetL2GO. There two possible reasons why the proposed method is superior to lnc-GFP and Lnc2Function. First of all, MPrNMF integrates multiple heterogeneous genomic data via the matrix factorization, which is more accurate to characterize lncRNA-ontology associations. Second, the multiple heterogeneous genomic data are regularized separately, rather than fusing them via a linear function. However, the proposed algorithm is inferior to NeuraNetL2GO. In detail, the Fmax for MPrNMF is 0.309, while that of NeuraNetL2GO is 0.336. There also two possible reasons. First of all, the MPrNMF algorithm is also a network-based method, requiring the networks are connected, which excludes away many lncRNAs or genes for analysis. The second reasons is that MPrNMF does not fully explore the topological information of networks, while the NeuraNetL2GO makes use of graph embedding features from networks.

**Figure 5:**
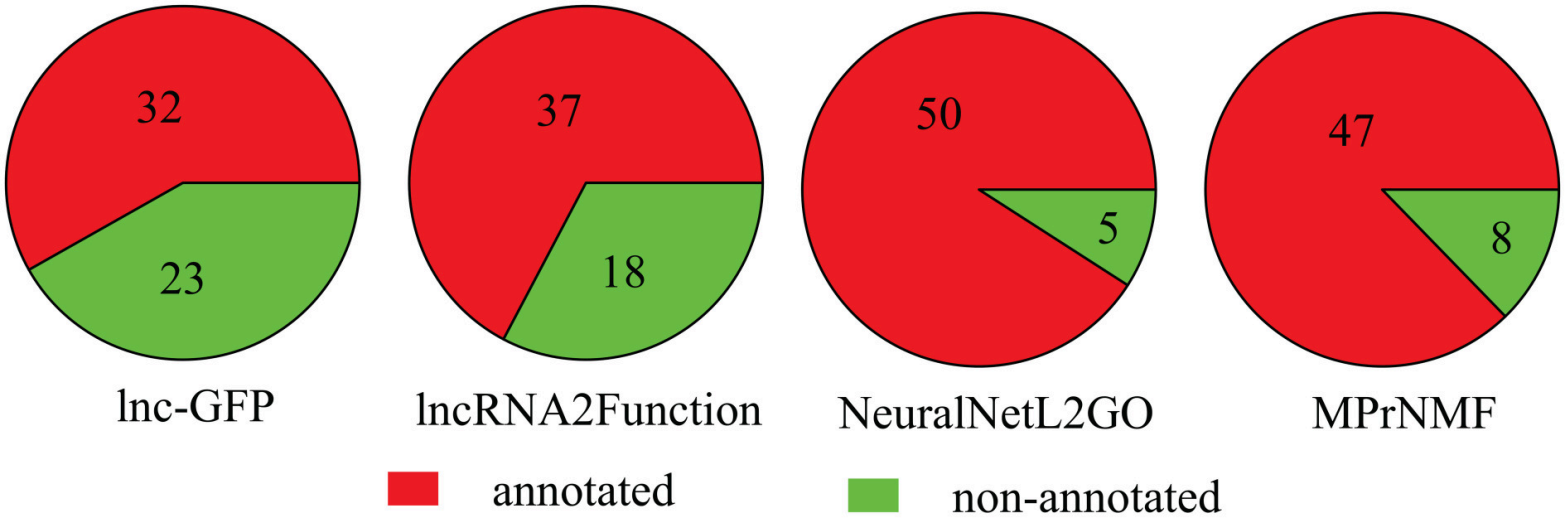
The number of lncRNAs that are correctly annotated by various algorithms.

Furthermore, we also compare these algorithms in terms of the number of lncRNAs that are annotated with a least one biological process GO term. As shown in Figure 5, 47 lncRNAs are correctly annotated by the proposed method, which is significantly higher than lnc-GFP and Lnc2Function. Even though it is not as high as that of NeuraNetL2GO, the difference is not significant (*p*-value = 0.387, Fisher Exact Test).

In MNrNMF, multiple views of lncRNA-gene associations are used. Then, we investigate the performance of each view of the associations. The Fmax of the proposed algorithm based on co-expression lncRNA-gene associations is 0.242, while that based on the disease lncRNA-gene associations is 0.278. These results indicate that the effective integration of heterogeneous genomic data is promising on the prediction of lncRNA-ontology.

### 4.5. Case Study

In this subsection, we apply MPrNMF to lncRNA instance to show the application of the proposed algorithm. HOTAIRM1 is an intergenic lncRNA between HOXA1 and HOXA2. Evidence shows that HOTAIRM1 is a critical regulator for the expression level of HOXA1 and HOXA4 (Zhang et al., 2009, 2014), which is involved in cell growth in leukemia cells. We apply the MPrNMF algorithm to predict the functions of HOTAIRM1, and it discovers 5 ontology functions: biological regulation, cellular process and signal transduction. These functions have been validated by the previous studies, indicating that the proposed method is applicable to predict the ontological functions of lncRNAs.

## 5. Conclusion

More and more lncRNAs have been identified in the past few years. However, the functions of vast majority of lncRNAs are poorly characterized. In this study, we propose a novel algorithm to predict the functions of lncRNAs via integrating multiple types of genomic data. The results demonstrate that the proposed algorithm is superior to the network-analysis based methods. However, the proposed method has some limitations. First, only the expression and disease data are used to construct the lncRNA-gene associations, which cannot fully characterize the relations. However to construct more reliable lncRNA-gene associations is promising in predicting the functions of lncRNAs. Second, the proposed method cannot fully make use the topological information in the multiple networks, such as graph embedding features. In the further studying, we will investigate how to solve these two issues.

## Author Contributions

JZ and XM designed the method and JZ coded the algorithm. JZ and XM wrote the paper.

### Conflict of Interest Statement

The authors declare that the research was conducted in the absence of any commercial or financial relationships that could be construed as a potential conflict of interest.
